# Supplementary material for: Ruthenium Complex HB324 Induces Apoptosis via Mitochondrial Pathway with an Upregulation of Harakiri and Overcomes Cisplatin Resistance in Neuroblastoma Cells In Vitro
Source: Int J Mol Sci. 2023 Jan 4;24(2):952. doi: 10.3390/ijms24020952 (PMC9866957; doi:10.3390/ijms24020952)
Supplement: Supplementary file 1 [file ijms-24-00952-s001.zip › ijms-2081788-supplementary.pdf]

Supporting information (SI)

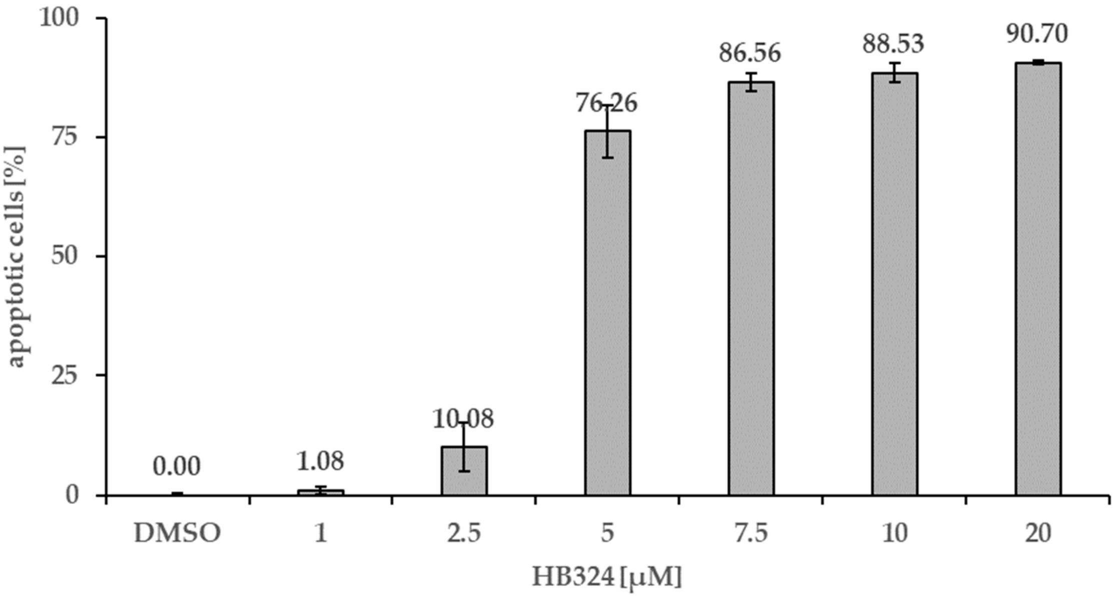

Figure S1 Nalm-6 cells were incubated for 72 h with increasing concentrations of HB324. Solvent treated cells (0.5 % DMSO) served as control. Nuclear DNA fragmentation was quantified by flow cytometric determination of hypodiploid DNA. Data are given in % hypoploidy (subG1), which reflects the number of apoptotic cells as mean values ± SD (n=3).

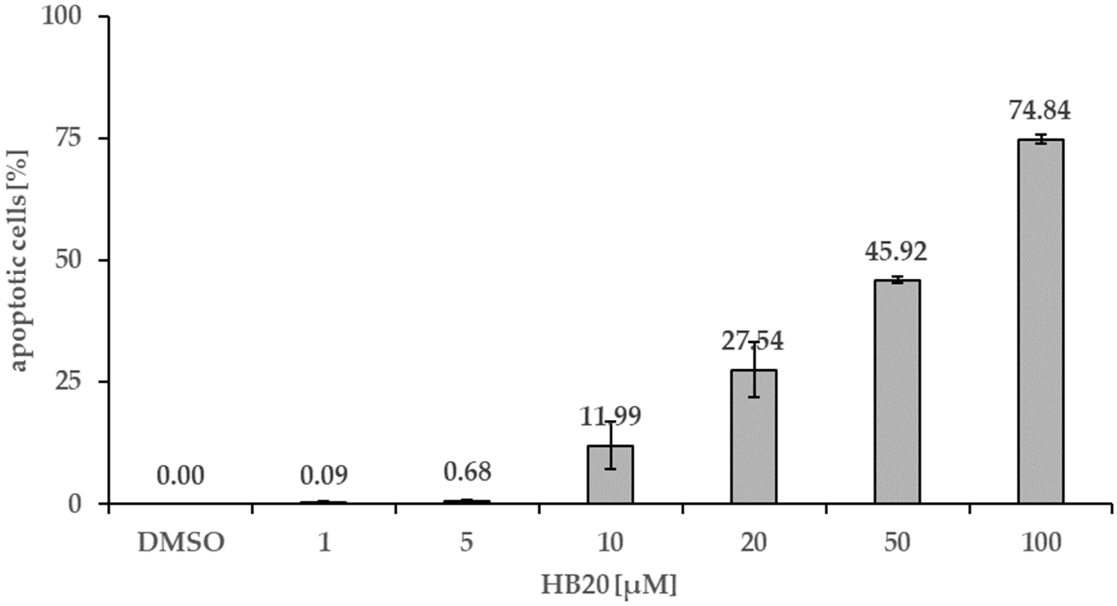

Figure S2 Nalm-6 cells were incubated for 72 h with increasing concentrations of HB20. Solvent treated cells (0.5 % DMSO) served as control. Nuclear DNA fragmentation was quantified by flow cytometric determination of hypodiploid DNA. Data are given in % hypoploidy (subG1), which reflects the number of apoptotic cells as mean values ± SD (n=3).

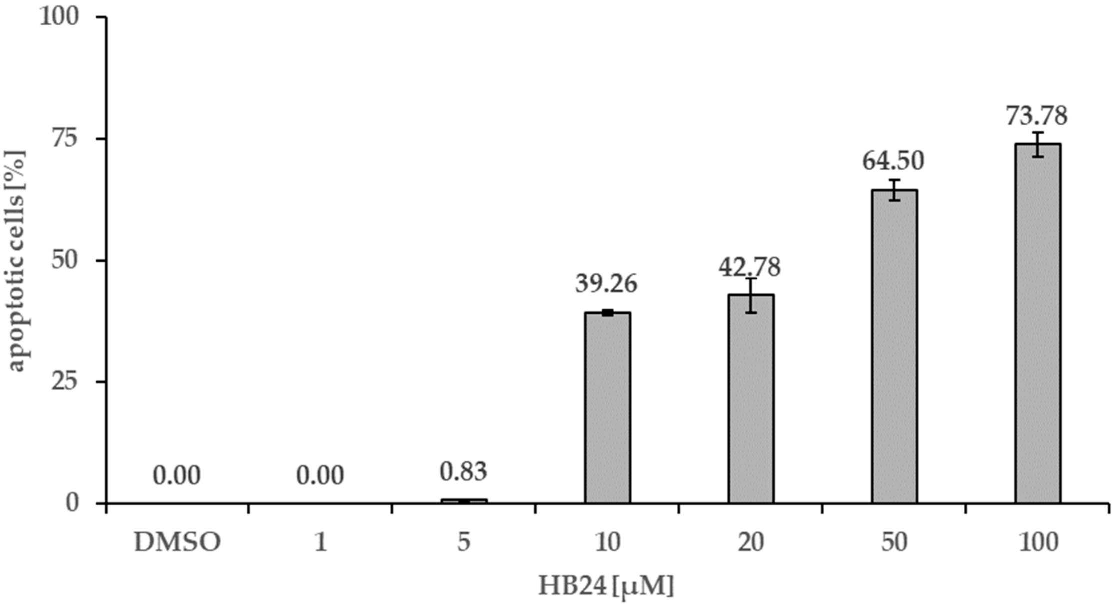

Figure S3 Nalm-6 cells were incubated for 72 h with increasing concentrations of HB24. Solvent treated cells (0.5 % DMSO) served as control. Nuclear DNA fragmentation was quantified by flow cytometric determination of hypodiploid DNA. Data are given in % hypoploidy (subG1), which reflects the number of apoptotic cells as mean values ± SD (n=3).

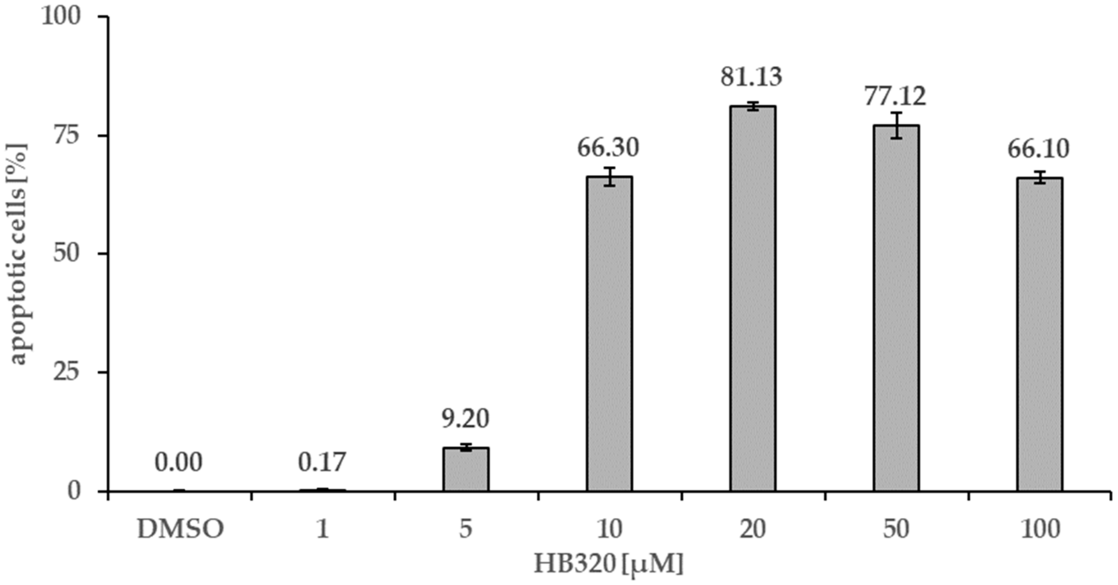

Figure S4 Nalm-6 cells were incubated for 72 h with increasing concentrations of HB320. Solvent treated cells (0.5 % DMSO) served as control. Nuclear DNA fragmentation was quantified by flow cytometric determination of hypodiploid DNA. Data are given in % hypoploidy (subG1), which reflects the number of apoptotic cells as mean values ± SD (n=3).

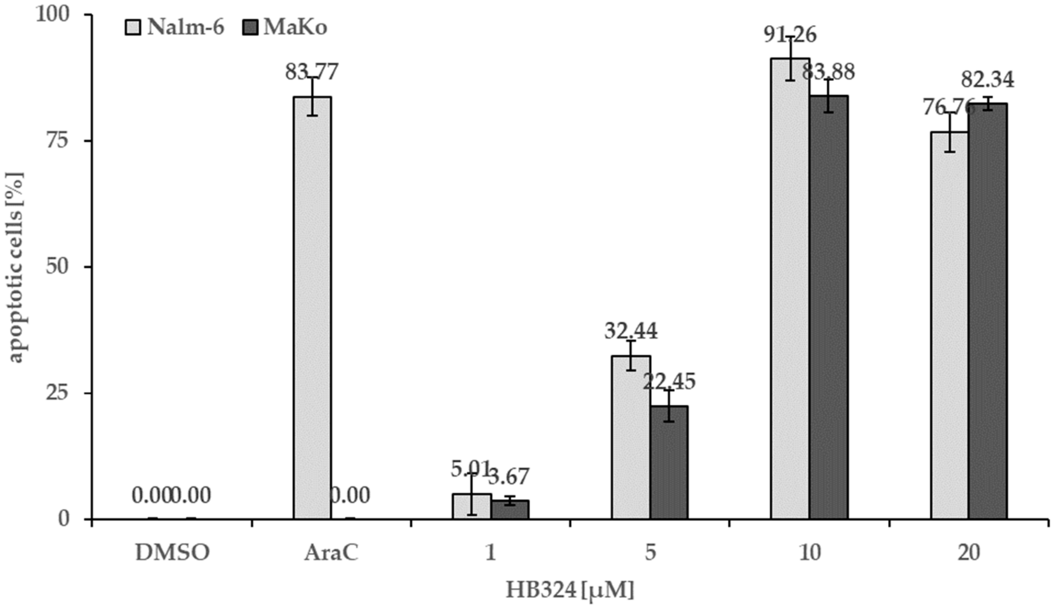

Figure S5 Apoptosis induction induced by HB324 in Nalm-6 and cytarabine-resistant Nalm-6 cells (MaKo). After 72 h of incubation with different concentrations of the agent DNA fragmentation was measured via FACS scan analysis. Solvent treated cells (0.5 % DMSO) served as control. 1.4 µM cytarabine (AraC) served as positive control. Values of DNA fragmentation are given as mean percentages of cells with hypodiploid DNA ± S.D. (n=3).

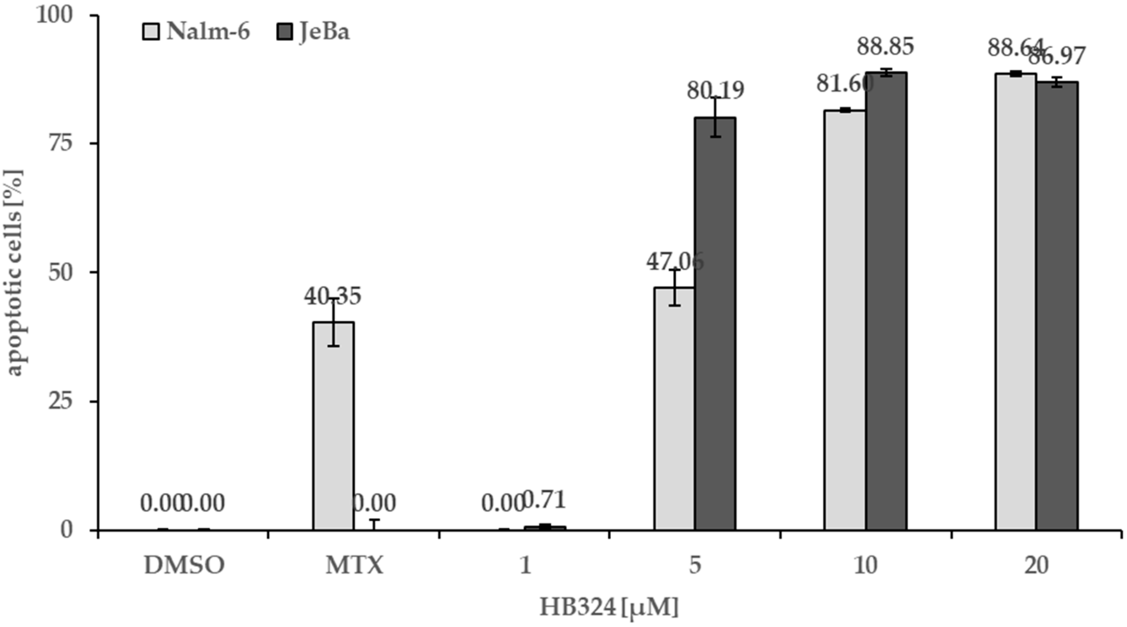

Figure S6 Apoptosis induction induced by HB324 in Nalm-6 and methotrexate-resistant Nalm-6 cells (JeBa). After 72 h of incubation with different concentrations of the agent DNA fragmentation was measured via FACS scan analysis. Solvent treated cells (0.5 % DMSO) served as control. 50 nM methotrexate (MTX) served as positive control. Values of DNA fragmentation are given as mean percentages of cells with hypodiploid DNA ± S.D. (n=3).

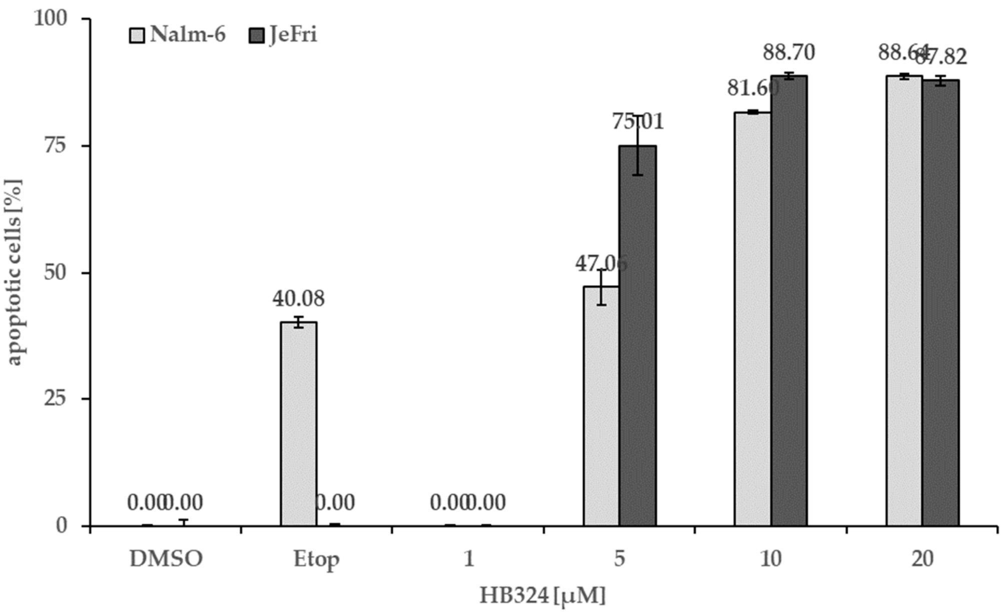

Figure S7 Apoptosis induction induced by HB324 in Nalm-6 and etoposide-resistant Nalm-6 cells (JeFri). After 72 h of incubation with different concentrations of the agent DNA fragmentation was measured via FACS scan analysis. Solvent treated cells (0.5 % DMSO) served as control. 0.36 µM etoposide (Etop) served as positive control. Values of DNA fragmentation are given as mean percentages of cells with hypodiploid DNA ± S.D. (n=3).

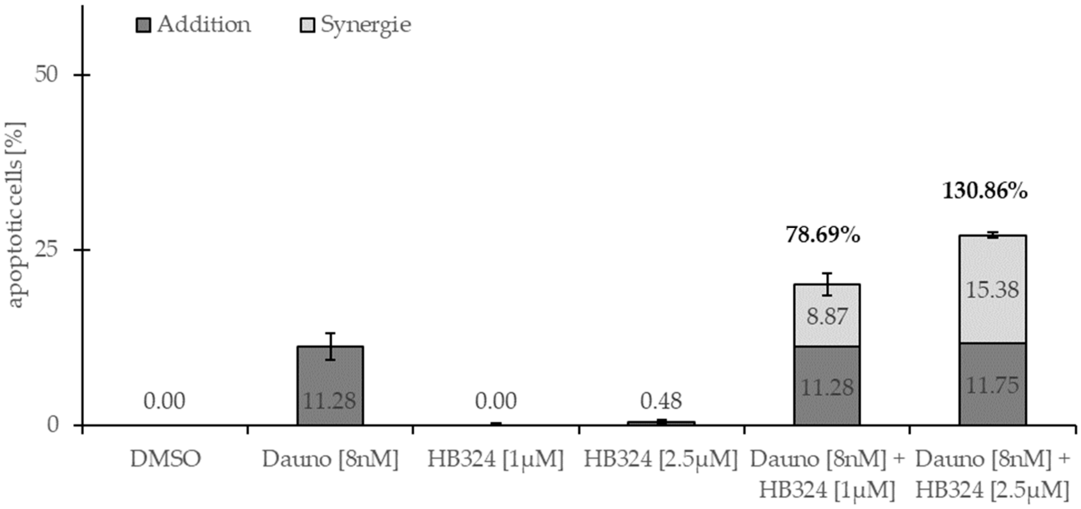

Figure S8 Nalm-6 cells were treated with 1 µM and 2.5 µM of HB324 and 8 nM of daunorubicin (Dauno) alone and in combination. Solvent treated cells (0.5 % DMSO) served as control. After 72 h of incubation, DNA fragmentation was measured by flow cytometric analysis. Values are given in percentage of apoptotic cells and as mean values ± SD (n=3). The synergistic effect is shown as the percentages of the additional number of apoptotic cells relative to the sum of the individual number of apoptotic cells.
